# Vision screening in pre-school children in Asmara, Eritrea

**DOI:** 10.1186/s12886-026-05206-9

**Published:** 2026-08-11

**Authors:** Samuel Tesfamichael Kibrom, Mengis Tesfamariam Fisshaye, Yohannes Solomon Ghebremedhin, Tsige Mnasie Smret

**Affiliations:** 1 Department of Optometry, Eritrean Air Force Hospital, Asmara, Eritrea; 2 Department of Optometry, Senafe Community Hospital, Senafe, Eritrea; 3 Department of Optometry, Biet Mekae Community Hospital, Asmara, Eritrea

**Keywords:** Vision screening, Pre-school children, Visual impairment, Preventable blindness

## Abstract

**Background:**

Preschool-aged children with vision problems are at a critical period of visual development and screening could identify and lead to treatments that could improve vision. So, this study conducted vision screening and identified the common vision and eye problems in this age group for early management.

**Method:**

Cluster sampling was used in selecting the regions of Biet Mekae and Villagio randomly to be the samples of the city. All the children from the two communities under the age of 5 years were included. Additionally, another set of tests were conducted on the children, who were referred for failing one or more of the tests to correctly diagnose their condition.

**Results:**

The study included 662 children, out of which 12.1% failed the tests and were referred for further investigation. Among those referred, 12.5% were found to be false positives, 26.25% were drop outs, and the rest 61.25% were diagnosed with different conditions. VKC was found in 10% of the referred children, allergic conjunctivitis in 21.25%, strabismus was found in 15%, bacterial conjunctivitis in 2.5%, corneal ulcer in 2.5%, NLDO in 3.75%, and the remaining 6.25% phoria, blepharitis, dacryocytitis.

**Conclusion:**

The results suggest that, vision problems and eye diseases which may lead to a more serious level of irreversible visual impairment later in life are common in pre-school children. Moreover, vision screening examination testability was highest in the age group 3–5 years, so interventions could be made more efficient by concentrating in this age group.

## Introduction

Vision is fundamental to a child’s early cognitive development and for learning new skills, making ocular health an essential component of overall pediatric well-being. Despite its high importance, childhood eye health remains underrepresented in global health agendas, particularly in developing regions. According to Borrel, children under-five years of age have the highest risk of blindness. Most blind children are either born blind or become blind before their fifth birthday [1]. Early detection and intervention are essential; because the infant visual system is immature and its neural pathways are not yet fully established, normal visual development depends heavily on unobstructed sensory inputs during critical early windows [2]. Deprivation of normal visual experience during this developmental period prevents the visual cortex from properly developing the capacity to have sharp vision.

Childhood blindness is strongly associated with poverty and child morbidity. In low-income countries with high under-5 years old morbidity and mortality rates, the prevalence of child blindness may be as high as 1.5 per 1000 children; while in high-income countries with low under-5 years old mortality rates, the prevalence of child blindness is around 0.3 per 1000 children [2]. Another matter of concern in developing countries is that up to 60 per cent of children die within a year of going blind [3, 4]). Researchers have identified a number of variables that are closely associated with poor child eye health and visual impairment, which include low socioeconomic status, inadequate access to adequate nutrition, limited access to clean drinking water and adequate sanitation facilities [5, 6]. In the African context, a lack of information and resources as well as recourse to traditional healing methods which are harmful to eye health, exacerbate the problem. According to the United Nations Development Program, although Africa has shown significant economic improvements over the last three decades, most countries in the region still have low levels of human development where access to quality healthcare is limited. Nevertheless, according to WHO, hundreds of thousands of children in Africa live with blindness, approximately 50% of which is entirely preventable or treatable through targeted interventions such as nutritional supplementation, immunization, refractive correction, and surgery [7]. Globally, the World Health Organization estimates that up to 80% of all blindness is preventable or treatable [8].

Despite the high manageability of these conditions, delayed presentation to specialized eye care centers frequently converts treatable conditions into permanent visual impairment. Furthermore, children under five are not matured enough to report or complain their visual problem or recognize normal visual baselines, making early identification and management difficult. Consequently, vision screening of every child in this age group becomes the only way of assessing any morbidity in the child’s ocular condition.

Vision screening comprises a rapid and basic assessment that helps correctly identify those who are in need of further examination. Pediatric vision screening specifically targets early identification to facilitate timely referral and management. As observed by Rocha et al., the spectrum of pediatric ocular conditions frequently includes allergic conjunctivitis, nasolacrimal duct obstruction, dacryocystitis, significant refractive errors, amblyopia, blepharitis, bacterial conjunctivitis, binocular vision anomalies, corneal ulcers, and vernal keratoconjunctivitis (VKC) [9].

Identifying and managing these conditions early on such sensitive developmental period offers a timely opportunity to prevent long-term visual disability. Accordingly, this study aims to investigate the prevalence of common eye and vision disorders among children aged birth to five years in Asmara, and to evaluate effective, context-appropriate screening protocols for this age group.

## Methods

### Study design, population and setting

This was a hospital-based cross-sectional study performed on all children of the age birth to five years of age in Biet Mekae and Villajo communities by a team of four optometrists. Cluster sampling was employed to select the population of Biet Mekae and Villajo communities to represent the population of Asmara. The study had two parts with the first part focusing on performing a cross-sectional screening of all the children of those communities, and the second part was conducting a thorough OPD examination to determine the cause of the failure in the vision screening. The study was conducted in Biet Mekae Community Hospital and a follow up examination was done in Brhan Ayni Referral Hospital in August, 2017.

### Sample size

All the children of the communities of Biet Mekae and Villajo were the samples used to represent the children of Asmara, so there was no sample size calculation but rather included all the children of our sample communities.

### Sampling techniques

The sampling technique that was employed was cluster sampling that selected the regions of Biet Mekae and Villagio randomly to be the samples of the city. All the children from the two communities under the age of 5 years were included.

### Inclusion and exclusion criteria

All children of the age birth to 5 years old who are residents of the above-mentioned communities (living in these areas for more than six months) were included. Children who weren’t permanent residents of the communities or who were above the age limit were excluded.

### Research tools and instruments

A data collection table was prepared, which included the demographic details of the child and the result of the tests which were performed on that child. The study adopted Kansas Vision screening guidelines 2004 as the screening protocol [10].

### Examination

In total 10 tests were conducted on the children, which were performed according to their age group. The tests were classified into mandatory and recommended for the age groups depending on their complexities, and this classification was adapted from Kansas Vision screening guidelines 2004 [10]. There were also two check-lists, i.e., ABC vision check-list and risk factors check-list, which were asked to the parents. In Table 1, the tests, their description and the age groups they were conducted on are listed.

**Table 1:** Tests and the recommended age groups they were conducted on

Vision screening test	Purpose	Pass criteria	Fail criteria	Birth to 6 months	6–18 months	18 months to 3 years	3–5 years
Eye lid reflex	presence of the protective blink reflex (gross VA)	blink in response to lowered hand	Blink absence in response to the lowered hand after two opportunities	P			
Risk factors(checklist)				P	R	R	R
Fixation	presence of a visual orienting response (gross VA)	fixating on the target with both eyes for at least 2 s	Failure to fixate on the target for 2 s	P	R	R	R
Tracking	oculo-motor development	Smooth, continuous movement with the eyes remaining in symmetrical alignment	monocular gaze only or failure to maintain gaze	P	P	P	P
Pupil response	neurological disorder	rapid, smooth constriction of the pupil when stimulated by the light, followed by smooth dilation in the absence of the light	sluggish, jerky, or asymmetrical	P	P	P	R
Corneal light reflex	Tropias	Light reflex in a similar position in both pupils	Light reflex in different position in both pupils	P	P	P	P
ABC’s of vision (checklist)				P	P	P	P
Cover-uncover test	Phorias and tropias	No movement of eyes upon uncovering	Movement of eyes upon uncovering		P	P	P
Near point of convergence	Convergence of eyes on near viewing	convergence and constriction of pupils to focus on moving object and a symmetrical response until object is within 3” from the bridge of the nose	poor fixation or asymmetrical response of eyes to the moving object beyond 3” or failure to converge to 3” from bridge of nose		P	P	P
Fusion test (worth 4-dot test)	Suppression or diplopia	Detection of four dots	Detection of 2,3, or 5 dots			P	P
Perception of depth (random dot)	stereopsis	Identification of raised card four times out of six attempts	Failure to identify raised card four times out of six attempts			P	P
Distance VA (lea chart)						P	P

P= priority for the age group

R= recommended if concern is present

### Data analysis

The obtained data were entered into SPSS version 20, and various statistical tests were run on the software. A confidence interval of 95% was taken and a p-value less than 0.05 was considered significant. Various descriptive tests were run to identify trends among the variables and describe the findings.

## Results

### Summary of demographic data

The study population was comprised of children aged between one month and 5 years. The total number of screened children was 662, out of these, 326 (49.2%) were males and 336 (50.8%) were females. The demographic distribution of the children across their age groups and their gender is presented in Table 2.

**Table 2:** Demographic distribution of the children

Groups	Age	Gender		Total
		Males	Females	
Group 1	Birth to 6 months	21	17	38 (5.74%)
Group 2	7–18 months	68	72	140 (21.14%)
Group 3	19–36 months	109	110	219 (33.08%)
Group 4	37–60 months	128	137	265 (40.03%)

### Results of post-screening investigations

In the screening process, 582 (87.9%) children passed the examinations while 80 children (12.1%) failed the tests. Those who failed the tests were referred for further investigation to determine the accurate diagnosis of their condition and do appropriate management. Among those 80 children, 21 of them dropped out of the study and never came back for the follow up exam, 10 children passed the test on the second examination and were categorized as false positive and 2 were referred to BARH for further examination. The diagnosis of the remaining 47 children upon further examination is presented in Table 3.

**Table 3:** Results of post-screening exams for those who failed the screening

Diagnosis	Group 1	Group 2	Group 3	Group 4	Total	Percentage among referred
VKC	0	0	0	8	8	17%
Allergic Conjunctivitis	0	2	5	10	17	36.2%
Strabismus	1	1	5	5	12	25.5%
Bacterial Conjunctivitis	0	1	1	0	2	4.3%
Corneal ulcer	0	0	1	1	2	4.3%
NLDO	0	2	1	0	3	6.4%
Phoria	0	0	0	1	1	2.1%
Blepharitis	0	0	0	1	1	2.1%
Dacryocystitis	0	0	0	1	1	2.1%
**Total**	**1**	**6**	**13**	**27**	**47**	**100%**

### Performances of children in vision screening tests and checklists of vision

Overall, there were 6048 tests planned to be conducted. Out of these, 4881(80.7%) were successfully performed with 4702(77.7%) passes and 179(3%) fails. The rest 1167(19.3%) were not performed. The more complex tests were performed on the older children and simple tests such as eye lid reflex test was performed on the first group only. The risk factors of vision and pupil response tests are recommended to be conducted on the younger age groups, but they were performed in age groups in this study for the reason of having complete tests. Table 4 shows the list of the tests performed along with their results for each age group.

**Table 4:** Performance on objective tests

	Pass	Fail	Not performed	Total
**ABCs of vision**				
Group 1	33	5	0	38
Group 2	120	20	0	140
Group 3	183	36	0	219
Group 4	228	36	1	265
Total	564	97	1	662
**Risk factors of vision**				
Group 1	35	3	0	38
Group 2	130	10	0	140
Group 3	197	22	0	219
Group 4	248	17	0	265
Total	610	52	0	662
**Hirschberg test**				
Group 1	31	1	6	38
Group 2	134	1	5	140
Group 3	210	3	6	219
Group 4	259	3	3	265
Total	634	8	20	662
**Pupil response test**				
Group 1	35	0	3	38
Group 2	138	0	2	140
Group 3	217	0	2	219
Group 4	260	2	3	265
Total	650	2	10	662
**Tracking test**				
Group 1	31	1	6	38
Group 2	127	0	13	140
Group 3	203	0	16	219
Group 4	259	2	4	265
Total	620	3	39	662
**Cover test**				
Group 2	85	1	54	140
Group 3	161	5	53	219
Group 4	254	5	6	265
Total	500	11	113	624
**NPC**				
Group 2	115	0	25	140
Group 3	194	0	25	219
Group 4	256	4	5	265
Total	565	4	55	624

### Performances on subjective tests

There were three subjective screening examinations for children who were above 18 months old (i.e. for groups 3 and 4). In the VA test, VA of 48.7% of the children in group 4 was measured while the rest 51.3% were not tested. In group 3, VA was measured in one child only. In Table 5 the performance of the children in the different subjective examinations is shown.

**Table 5:** Performance on subjective tests

Visual Acuity						
	6/10	6/7.5	6/6	Not performed	Pass	Fail
Group 3	0	0	1	218	1	0
Group 4	12	37	80	136	129	0

	**Pass**			**fail**	**Not performed**	
**Stereo test**						
Group 3	2			0	217	
Group 4	187			2	76	
**Worth 4 dot**						
Group 3	4			0	215	
Group 4	219			0	46	

## Discussion

### Demography and testability

This study provides the first data on preschool vision screening in Asmara’s communities. The demographic distribution of the children was almost equal number of female and male children, with a slightly higher number of females than males, with 50.8% and 49.2% respectively. Out of all the tests which were planned to be performed, 80.3% were successfully performed, which is little more than a similar preschool screening conducted in USA which reported 77% of testes performed. Meanwhile in the data reported from African Americans and Hispanics, 83% and 84% of the children had their VA successfully measured respectively [11], in this study however, only in 48.7% of the children aged 3 to 5 years was VA successfully measured, while only 1 child had VA measured in the age group 19–36 months. This low testability is due to lack of an appropriate VA chart. This in turn has caused a failure to detect vision and vision development problems in the study population.

### Common vision and eye problems

The prevalence of visual and various eye conditions was found to be lower than the observation in most of other similar studies conducted throughout the world. The observation of VKC in this study was 1.2% of all the total study participants, which was lower than a 6.7% prevalence found in a cross sectional study in northern Nigeria which was conducted in children aged 4–15 years. The prevalence of ocular infections was 0.7%, again lower than the finding in the Nigerian study which found 1.3% of ocular infections [12]. Another study in a school located in northern Nigeria showed a 7.3% prevalence of allergic conjunctivitis in a study which was conducted in children aged 5–17 years, while in this study the prevalence was 2.6% [13].

Strabismus had 1.8% prevalence in this study similar to a prevalence of 1.5% in a study in preschool study American children. In general, population-based studies have reported a prevalence of strabismus ranging from 2% to 5% among preschool and school-aged children in Caucasian children [14]. In a study in Baltimore in preschool aged children found a strabismus prevalence of 3.3% in white and 2.1% in African American children [14].

Fewer than 20% of children receive adequate screening. Today, many eye diseases go undetected at stage where intervention would have otherwise been effective. As a result, amblyopia remains the most common cause of monocular visual impairment among children, as well as among young and middle-aged children [11].

Refractive error is one of the amblyogenic factors and amblyopia in return can also worsen refractive error. As result a proper identification and management of the problem is mandatory in the visual development and performance of a child. However, since the measurement of subjective visual acuity in the younger ages is difficult, the condition remains untested in many cases. In Multi Ethnic Pediatric Eye Disease Study, 48-59-month-old children consistently showed more Visual Impairment than the older age group. On the other hand, VI and amblyopia were low in the young children because of the lower testability in the younger age group, meaning VI may simply have gone undetected. There was more VI in higher age groups, reflecting improved test performance in older children [16].

The Kansas vision screening guideline 2004 recommends the use of an HOTV, Lea or Snellen’s charts in screening preschool children [10]. However, in this study due to the unavailability of these charts, a picture chart was used and since most of the young children were unable to understand, there was low VA testability. Therefore, the prevalence of amblyopia and refractive error was very low compared to other studies. According to this research there was 0% incidence of amblyopia in all the children. This data was very different when compared with the data found from previous reports on amblyopia in preschool children from African- Americans 2.6%, Hispanics 1.5%, Australia 0.9%, South Africa 0.3% and Chile 1.4% () [17–21]. 

The prevalence of refractive error was 0.15% in this study, which was also very low when compared to the reports from Nepal 1.6%, China 29.9%, Rural India < 0.6%, South Africa < 0.6%, African-Americans2.2% and Hispanics 2.4% [20–22, 10, 18]. As mentioned in MEPEDS, it is obvious that the detectability of visual impairment and amblyopia is low at young age when compared to older age due to decreased testability.

According to Donahue et al. instrument-based screening detects amblyopia, high refractive error, and strabismus the best, it is particularly more useful in above 18-month-old children. As this study didn’t have these instruments, the VI and amblyopia were lower than most other studies.

### Testability

Tests which require certain level of subjective responses are more sophisticated examinations as they reveal the actual visual capability of a child. For the tests to be conducted, a child is needed to speak or give other advanced type of response. Therefore, the older the child is, the more responsive he/she becomes. This study was not far from this fact, children, starting from around 3 years of age, were having an increasing level of VA testability.

## Conclusion

Screening of pre-school children revealed 7.4% prevalence of ocular morbidities in children in these communities. Strabismus, which is a strong risk factor for amblyopia, was present in 1.8% of them. This may lead to irreversible VI if proper management is not made early on.

The age sub group appropriate for vision screening in this age group was from three to five years old. The vision screening procedures performed in the younger ages didn’t lead to proper diagnosis in most cases.

Another crucial observation is that most parents don’t take their children for vision screening, so having scheduled vision screening is beneficial for these children, and having appropriate charts like HOTV and Lea symbol chart is essential for these screenings.

## Limitations of the study

Age appropriate VA charts recommended by the guideline such as HOTV, Lea charts were not used because of their un-availability in the setup. As a result, the testability of VA was low.

Because of limitation of resources teller acuity cards, HOTV charts, photo refraction and Titmus fly tests, which were part of the Kansas Vision screening guideline were not performed.

## Appendix

### Risk factors — checklist

- Premature birth (4 weeks or more).
- In a neonatal intensive care unit for greater than four days, plus oxygen.
- Very low birth weight [< 1500 g (3.3 lbs.)]
- Birth defects involving the head or face.
- Family history of childhood vision impairment (i.e., amblyopia, severe refractive error, strabismus or any visual perception problems).
- Suspected of having a congenital infection (i.e., cmv, toxoplasmosis, maternal venereal conditions, rubella).
- Condition which is associated with vision problems (ex. down syndrome, cerebral palsy, hearing impairment, diabetes, juvenile rheumatoid arthritis, hydrocephalus, retinoblastoma).
- Congenital deafness (ex. usher syndrome).
- Parent, child or caregiver has concerns about their vision.
- Head trauma (ex. shaken baby syndrome).
- Brain tumor.
- Chemical exposure (ex. Lead poisoning, drugs during pregnancy, fetal alcohol syndrome).

### ABC’S of vision difficulty

This list can be used to identify the need for a vision examination and should be shared with the classroom teacher, parents, Infant-Toddler network, or child’s physician.

### A’S - Appearance of the eyes

- Reddened eyes
- Watery eyes
- Encrusted eyelids
- Frequent stye
- Droopy eyelids
- Eyes in constant motion, eyes jerk on fixation
- Eyes crossed- turning in or out at any time (> 6mos.)
- Difference in size of eyes

### B’S - Behaviors indicative of possible vision difficulty

- Abnormal sensitivity to light
- Rubs eyes frequently
- Blinks excessively or rarely blink
- Stares at bright lights frequently
- Turns head so as to use one eye only to look at object in front of child
- Brings objects up to one eye rather than both eyes to view it
- Covers or closes one eye frequently
- Tilts head to one side when looking at object in front of child (> 6 months)
- Exhibits poor eye-hand coordination (> 6 months)
- Doesn’t appear interested in looking at toys or faces (< 6 months)
- Doesn’t respond to visual toys but is responsive to toys with sound (3 to 12 months) − Stares at own hand or an object and/or moves own hand or object in front of the eyes frequently (> 12 months)
- Places an object within a few inches from the eyes to look at it (> 18 months)
- Thrusts head forward or backward while looking at distant objects
- Squints, frowns or scowls when looking at objects (or reading)
- Have academic difficulties
- Avoids close work
- Exhibits short attention span
- Exhibits “acting out” or “class clown” behavior
- Dislikes tasks requiring sustained visual concentration
- Exhibits nervousness, irritability or restlessness after maintaining visual concentration
- Exhibits unusual fatigue after completing a vision task
- Places head close to book or desk when reading or writing
- Loses place while reading
- Uses finger to keep place while reading
- Says aloud or mouths the words while reading
- Moves head rather than eyes while reading
- Exhibits difficulty remembering what is read
- Persistent letter reversals after the second grade
- Confuses similar words when reading
- Confuses the following letters when reading or spelling: o and a; h and n; e and c; n and m; f and t
- Exhibits unusual awkwardness

### C’s - Complaints associated with using the eyes

- Headaches.
- Nausea and/or dizziness.
- Eyes hurt.
- Burning, itching, or tearing of eyes.
- Blurring or double vision.

## Data Availability

The data-set supporting the findings of this research are available from the corresponding authors.

## Abbreviations

USPSTF: United States Preventive Services Task Force
VA: Visual Acuity
VEP: Visually Evoked Potential
ROP: Retinopathy of Prematurity
NLDO: Nasolacrimal Duct Obstruction
VKC: Vernal Keratoconjuctivitis
PEE: Punctate Epithelial Erosion
LogMAR: Logarithmic Minimal Angle of Resolution
BARH: Birhan Ayni Referral Hospital
BMCH: Biet Mekae Communnity Hospital
VI: Visual impairment
MEPEDS: Multi Ethic Pediatric Eye Disease Study
